# APAAACI 2023 International Conference: The innovation revolution in allergy, asthma, and immunology

**DOI:** 10.5415/apallergy.0000000000000130

**Published:** 2023-12-08

**Authors:** Bernard Yu-Hor Thong, Ruby Pawankar

**Affiliations:** 1 Department of Rheumatology, Allergy and Immunology, Tan Tock Seng Hospital, Singapore; 2 Department of Pediatrics, Nippon Medical School, Nippon Medical School, Tokyo, Japan

After more than a decade since the Asia Pacific Association of Allergy, Asthma and Clinical Immunology (APAAACI) 2010 congress was last hosted in Singapore, the conference was held again in Singapore from October 23, 2023 to October 26, 2023, at the same iconic Suntec International Exhibition and Convention Centre. Healthcare transformation in the delivery of care for children and adults with allergic and immunologic diseases was rapidly accelerated during and following the coronavirus disease pandemic with the use of digital health and telemedicine [1-3]. Embedded within APAAACI 2023’s 3 plenary, 26 symposia, 4 workshops, and 8 oral and poster presentation sessions were a few overarching themes: a call for more multi-, inter-, and transdisciplinary clinical, education, and research collaborations; increase in the proportion of Asian representation (since Asia represents more than 50% of the world’s population) in clinical trials and real-world data; and the use of big data and analytics to link up the world’s allergy/immunology centers of excellence [4].

With APAAACI’s strong commitment to addressing the global issues of climate change and air pollution on health that are major issues in the Asia Pacific region [4-7], the conference was held as a sustainability event with a keynote lecture on the G20 India declaration on lifestyle and environment and an inaugural speech highlighting Singapore Green Plan 2030 and Healthier SG as key elements of the conference. This was followed by a plenary session on “Environment and One Health” highlighting the environmental impact of climate change on allergies and noncommunicable diseases, the epithelial barrier hypothesis, and the one health approach to climate mitigation including a call to action in line with the G20 India 2023 declaration on climate change and one health [4, 5].

With a conference theme on the Innovation Revolution in Allergy Asthma and Immunology, comes the role of artificial intelligence (AI). AI is the simulation of human intelligence processes by machines, especially computer systems. AI has increasingly been used in health care. Given the capacity of AI to handle large data and complex relationships between variables, AI is well suited for applications in health care. The domains of AI include machine learning (ML), deep learning (DL), and natural language processing (NLP). ML refers to the process of developing systems with the ability to learn from data and make predictions without prespecified programming. DL is a subset of ML that uses layers of neural networks to process complex data. NLP is a method of computer-based analysis of unstructured text, for example, data mining from clinicians’ notes [8]. Generative AI describes algorithms that can be used to create new content, including audio, code, images, text, simulations, and videos, for example, ChatGPT (GPT stands for generative pretrained transformer). Artificial general intelligence (AGI), on the other hand, refers to a theoretical state in which computer systems will be able to achieve or exceed human intelligence. As of October 19, 2023, the US Food & Drug Administration has approved 171 AI-/ML-enabled medical devices, mostly in radiology and cardiology [9]. No device has been authorized that uses generative AI or AGI or is powered by large language models. Similar trends are observed in Conformité Européene-marked medical devices incorporating AI within the European Union [10]. More recently, AI has also been applied to allergy research in asthma, atopic dermatitis, rhinology, adverse reactions to drugs and vaccines, food allergy, anaphylaxis, urticaria, and eosinophilic gastrointestinal disorders [8,10]. In future, AI will improve our understanding of patient profiles, disease mechanisms, risk stratification, and precision diagnosis and aid in the management of allergic and immunologic disorders. Tutorials to harness electronic medical records (EMR) and AI will help clinicians improve their diagnostic and therapeutic capabilities, improve efficiency within the healthcare system, support physician work and lifestyle preferences, and reduce time spent on EMR [11]. However, given that elements of bias, ethics, and harm may be inappropriately incorporated at many levels of AI and ML modeling, there are many regulatory, governance, privacy, data, and legal considerations that need to be considered for patient safety before AI can be implemented as standard of clinical care within a specialty [8].

Personalized (precision) medicine in allergic and immunologic disorders encompass precision diagnosis, treatment, and prevention. Biomarkers are crucial for precision medicine, which help to define disease endotypes, clusters, precision diagnoses, identification of therapeutic targets, and monitoring of treatment efficacy. Powerful omics technologies together with AI approaches can help identify potential clinically useful biomarkers that can be accurately quantified using robust and reproducible methods. A large fraction of allergic diseases is characterized by a type 2 immune responses involving Th2 cells, type 2 innate lymphoid cells, eosinophils, mast cells, and M2 macrophages. Promising biomarkers of Th2 allergic diseases include sputum eosinophils, serum periostin, and exhaled nitric oxide (FeNO), for instance, in phenotyping and endotyping Th2 high asthma. Other biomarkers, for example, proinflammatory mediators, microribonucleic acids, eicosanoid molecules, epithelial barrier integrity, and microbiota changes, are also useful for diagnosis and monitoring of allergic diseases and can be quantified in serum, body fluids, and exhaled air [12]. The exponential growth of precision diagnostic tools, including omic technologies, molecular diagnostics, sophisticated genetic and epigenetic editing, imaging and nanotechnologies, have resulted in vast amounts of unbiased data enabling in-depth disease characterization [13]. The intelligent and appropriate use of these enablers will facilitate more precise diagnostic and therapeutic approaches for the increasingly complex allergy/immunology patients.

As a result of the development of immunogenomics, bioinformatics, and AI, new disease endotypes have been identified for various allergic and immunologic disorders following the discovery of new biomarkers, pathogenetic and metabolic pathways, and pathogenic genetic variants. Current disease taxonomy has had to be revised for better categorization. Thus, the European Academy of Allergy Asthma and Clinical Immunology (EAACI) 2023 paper on the Nomenclature for allergic diseases and hypersensitivity reactions [14] updates the EAACI 2001 [15] and World Allergy Organization 2003 nomenclature [16]. Hypersensitivity reactions originally described by Gell and Coombs have been extended into 9 different types comprising antibody (I-III), cell-mediated (IVa-c), tissue-driven mechanisms (V-VI), and direct response to chemicals (VII). Types I-III are linked to classical and newly described clinical conditions. Types Iva-c are specified and detailed according to the current understanding of T1, T2, and T3 responses. Types V-VI involve epithelial barrier defects and metabolic-induced immune dysregulation, and type VII involve direct cellular and inflammatory responses to chemicals. In clinical settings, several combinations of mixed types of hypersensitive reactions may coexist.

Similarly, the 2022 update on the classification of Human Inborn Errors of Immunity (IEI) from the International Union of Immunological Societies (IUIS) Expert Committee [17] categorizes IEI (what used to be termed “primary immunodeficiency diseases” or PID) into 10 tables, with subtables segregating groups of disorders into overlapping phenotypes using a combination of genetic, molecular, cellular, and immunological mechanisms of disease to define each IEI. When we contrast this with the IUIS classification of PID published in 1999 [18], it becomes obvious how the “innovation revolution” has advanced our specialty in the past 2 decades, and how it will continue to transform how we manage our patients, our clinical practice, and our daily lives.

## Conflicts of interest

The authors have no financial conflicts of interest.

## Author contributions

Writing: Bernard Yu-Hor Thong, Ruby Pawankar.
